# Double effects of mitigating cyanobacterial blooms using modified clay technology: regulation and optimization of the microbial community structure

**DOI:** 10.3389/fmicb.2024.1480069

**Published:** 2024-11-05

**Authors:** Jianan Zhu, Zhiming Yu, Xihua Cao, Wenbin Jiang, Liyan He, Xiaomiao Zang, Xiuxian Song

**Affiliations:** ^1^CAS Key Laboratory of Marine Ecology and Environmental Sciences, Institute of Oceanology, Chinese Academy of Sciences, Qingdao, China; ^2^Laboratory for Marine Ecology and Environmental Science, Qingdao Marine Science and Technology Center, Qingdao, China; ^3^University of Chinese Academy of Sciences, Beijing, China

**Keywords:** cyanobacterial bloom, modified clay, bloom control, phytoplankton community structure, function of microbiome

## Abstract

Harmful algal blooms (HABs) are global hazards under global climate change and eutrophication conditions. Modified clay (MC) method is widely used to control HABs in Asian and American coastal waters. However, little research has been conducted on the underlying mechanisms by which MC controls blooms in freshwater environments. Herein, experiments and bioinformatics analyses were conducted for MC-based control of freshwater blooms in a closed water body with an area of approximately 240 m^2^ in the Fuchun River, China. Results revealed that the dominant bloom species were *Microcystis*, and an 87.68–97.01% removal efficiency of whole algal biomass was achieved after 3 h of MC treatment. The weaker zeta potentials of *Microcystis* species and hydrophilic groups such as O-H and P-O-P in the extracellular polymeric substances (EPS) surrounding *Microcystis* cells made them easier to be flocculated and removed by MC particles, and the relative abundance of *Microcystis* decreased to 29.12% and that of *Cyanobium* increased to 40.97%. Therefore, MC changes the cyanobacterial community structure, which is accompanied by the elimination of *Microcystis* sp. apical dominance and enhanced competition between *Cyanobium* and *Microcystis* in the phytoplankton community, increasing cyanobacterial community diversity. Under MC treatment, residual microorganisms, including cyanobacteria, had a high potential for DNA damage repair and were more likely to survive after being subjected to oxidative stress. In the meanwhile, the abundance of genes involved in genetic information processing, signal transduction, and photosynthesis was decreased indicating that the residual microbiome was week in proliferation and light energy harvesting. Therefore, accompanied with the destruction of *Microcystis* colonies, MC changes the function of cyanobacteria and phycosphere microbiome, further hindering bloom development. These findings illustrate that MC can regulate and optimize the microbial community structure through which MC controls cyanobacterial blooms in ecosystems.

## Graphical abstract

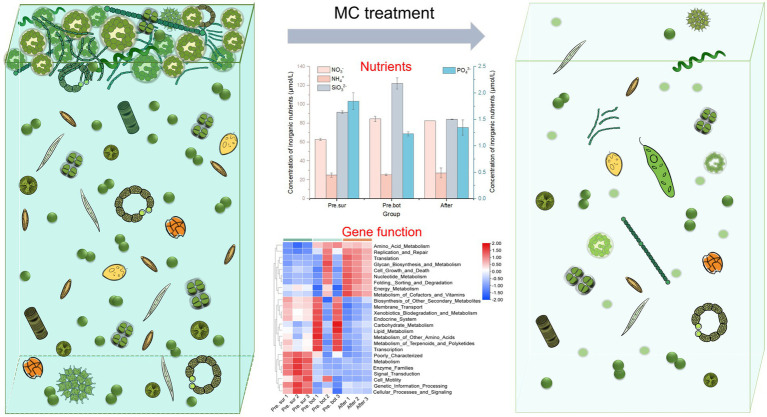

## 1 Introduction

Due to rapid industrial and agricultural development, as well as increased urbanization, large amounts of nutrient-rich waters are discharged into rivers, lakes, and seas, which is followed by vigorous phytoplankton proliferation and frequent severe bloom events (Anderson et al., 2002; Berry et al., 2017; Glibert et al., 2018; Heisler et al., 2008; Keck and Lepori, 2012). Recently, harmful algal blooms (HABs) have emerged as global hazards (Anderson et al., 2012; Yu et al., 2017). Blooms in freshwater are caused mainly by cyanobacteria, and a long hydraulic residence time and calm surface waters can provide the right conditions for the growth of HAB species (Paerl et al., 2011; Ren et al., 2020). Some cyanobacteria can release toxic secondary metabolites (e.g., *Microcystis aeruginosa* and *Planktothrix agardhii*), which are harmful to human health and ecosystem stability (Jang et al., 2003; Kurmayer et al., 2016). Therefore, choosing an appropriate control method for reducing the frequency, duration, and scale of HABs is a popular topic.

Theoretically, the most effective way to manage cyanobacterial blooms is to control phosphorus (P) and nitrogen (N) inputs to reduce water eutrophication (O'Neil et al., 2012; Qin et al., 2020). However, controlling blooms at the source is difficult because there are many sources of nutrient pollution. It is more feasible to achieve control at the terminal end with the help of physical, chemical, and biological methods (Gallardo-Rodríguez et al., 2019). For example, chitosan can lead to the flocculation and sinking of cyanobacteria and the lysis of cells during lake restoration (Mucci et al., 2017), and hydrogen peroxide (H_2_O_2_) and UV-C radiation at sufficient doses can also kill cyanobacteria (Spoof et al., 2020; Wang et al., 2015). Soil or sand surfaces modified with molecules such as chitosan or *Moringa oleifera*-derived coagulant have shown good results in cyanobacterial bloom management via enhanced flocculation and cell lysis (Li and Pan, 2015; Li and Pan, 2013; Wang L. J. et al., 2016). Clay is a natural mineral, its composition is more uniform and stable than that of other soil particles, and its particle size is smaller than that of sand, making it an ideal material for the flocculation of algal cells. Yu et al. (1994) proposed modified clay (MC) technology for HAB control, which involves modifying the surface of clay particles with polyaluminum chloride (PAC) to change the surface charge from negative to positive, greatly improving algae removal efficiency. Due to its advantages of being nontoxic, inexpensive, and highly efficient, MC has been widely used for HAB treatment in offshore China, and its use has been extended to many countries, such as the United States, Chile, Peru, and Malaysia (Devillier et al., 2024; Yu et al., 2017). The mechanisms by which MC controls HABs have also been investigated in marine microalgae, including mitigating most HAB biomass via flocculation and inhibiting the growth of residual algae via oxidative stress and programmed cell death (PCD) (Jiang et al., 2023; Liu et al., 2018; Zhu et al., 2018, 2019). In aquaculture water, the MC method was used to regulate shrimp-culture water quality, and the results revealed that MC could increase the diversity and stability of the microbial community structure in the water (Ding et al., 2021). In fact, the first field application of MC was for the treatment of cyanobacterial blooms in Xuanwu Lake, Nanjing, in 2005, which ensured the progression of the aquatic events of the 10th National Games of the People’s Republic of China. However, little research has been conducted on the mechanisms underlying the success of MC treatment of freshwater blooms.

In China, the Fuchun River has historical and cultural value, with a beautiful environment for tourism and rich fishery resources. It is also one of the source regions for the endangered species *Tenualosa reevesii*, as well as the fishery species *Hypophthalmichthys molitrix*, *Aristichthys nobilis*, *Lateolabrax japonicus*, *Hemisalanx prognathous* and *Eriocheir sinensis*. In 2023, cyanobacterial blooms were observed in this river in the Fuyang County section. We tested the effectiveness of MC in controlling cyanobacterial blooms in a ship channel that was confined to one side of the Fuchun River. Traditional biological and chemical research methods combined with high-throughput sequencing technology were used to investigate the effects of MC on the levels of various nutrients in the water and on the structure and function of both eukaryotic and prokaryotic phytoplankton communities. This study illustrates the mechanisms through which MC controls cyanobacterial blooms in fresh water and provides scientific evidence for the further promotion and application of MC to control HABs.

## 2 Materials and methods

### 2.1 Experimental design

**Figure 1:**
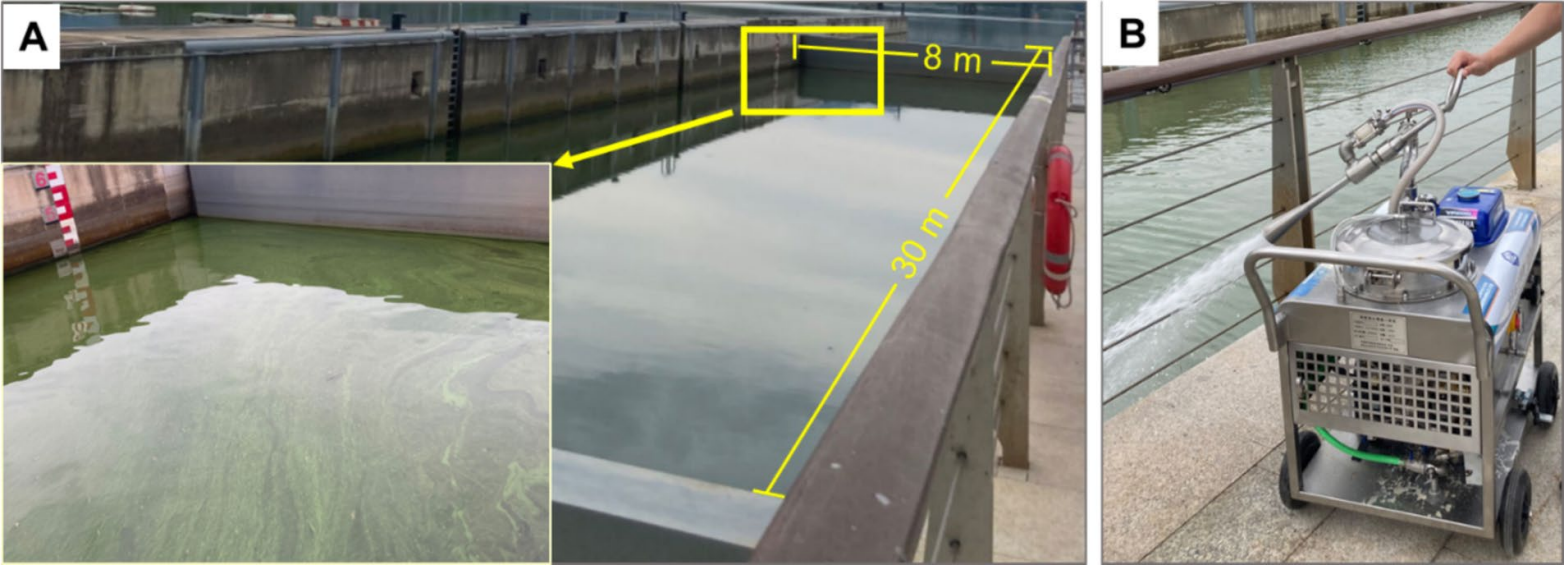
The experimental site (A) and the modified clay spraying method used to mitigate algal blooms (B).

The experiment was conducted in a region located on the southern side of the Fuchun River, approximately 2.5 km upstream of the water sports center in Fuyang District, Hangzhou. This region is a channel used for ship navigation, and a closed water body with an area of approximately 240 m^2^ and a depth of 4 m is formed when the channel is closed (Figure 1A). Algal bloom treatment was carried out in the above natural water via special MC spraying equipment (Figure 1B), and the amount of MC used was 230 kg.

### 2.2 Modified clay preparation

The MC used in all the experiments was an industrial product. The neutral clay material was a kaolinite produced in Hebei Province; the modifiers used were PAC and peroxymonosulfate (PMS) (its composition was 2KHSO_5_·KHSO_4_·K_2_SO_4,_ and the active ingredient was KHSO_5_). Therefore, the MC species used in this study was PAC-PMS-clay, or MC for short. The specific processes of MC preparation were as follows: kaolinite and PAC were mixed at a ratio of 5:1 by mass in a reactor, diluted in deionized water, stirred thoroughly, aged for 24 h at room temperature, filtered, dehydrated, and desiccated. Finally, the PAC-clay and PMS were mixed at a ratio of 20:3, producing the MC for spraying.

### 2.3 Phytoplankton sample collection

Phytoplankton community samples were collected before and after MC disposal. During the bloom, the majority of the biomass floats on the surface of the water. Considering that the vertical distribution of phytoplankton was not uniform, before MC treatment, phytoplankton samples were collected from both surface and bottom waters. After MC treatment for 3 h, there was no significant aggregation of algae on the water surface. Therefore, phytoplankton samples were collected from only one layer at a depth of 2 m below the surface water. Waters were sampled with a surface water sampler. The phytoplankton in the experimental waters was dominated by cyanobacteria, which are difficult to count and identify due to their small size and cell aggregation. To evaluate the community structure well, high-throughput sequencing was applied in this study for species identification and relative abundance analysis for both eukaryotic and prokaryotic phytoplankton. For each sample, 50–200 mL of water was filtered through a 0.22 μm pore-size isopore filter (Millipore, Germany) (Lu et al., 2020). These filters were placed in 2 mL cryogenic vials and flash-frozen in liquid nitrogen prior to transport to the laboratory, where the samples were stored at −80°C until DNA was extracted for analysis (Liu et al., 2022).

### 2.4 Measurement and analysis of the structure and function of the phytoplankton community

Experienced DNA extraction, PCR amplification (18S rDNA V4 region for eukaryotic phytoplankton, 16S rDNA V3 and V4 regions for prokaryotic phytoplankton), purification, and quality testing (using a NanoDrop 2000 spectrophotometer (Thermo Fisher Scientific, USA) and agarose gel electrophoresis), and all qualified samples were used to construct the sequencing libraries. Finally, an Illumina NovaSeq 6000 platform was used to carry out paired-end sequencing. Paired-end raw reads were merged via FLASH (v1.2.7)^1^ (Magoc and Salzberg, 2011). After filtering, clean tags were obtained. Dereplication by DADA2 (Callahan et al., 2016), amplicon sequence variants (ASVs) were used to analyze the phytoplankton species in each sample (Amir et al., 2017). Classify-sklearn of QIIME2 was applied for species annotation for further analysis of community richness, composition, and diversity at different classification levels (kingdom, phylum, class, order, family, genus, and species) (Bokulich et al., 2018). Alpha diversity indices, such as Chao1, Observed ASVs, Shannon and Simpson indices, were used to assess phytoplankton within the communities (Li et al., 2013). In addition, principal co-ordinates analysis (PCoA) based on unweighted UniFrac distances was carried out between different communities (Lozupone et al., 2011; Minchin, 1987). The bioinformatics package Phylogenetic Investigation of Communities by Reconstruction of Unobserved States (PICRUSt) is a useful tool for analyzing microbial function and has been widely used (Fei et al., 2020; Langille et al., 2013). In this study, PICRUSt was used to evaluate the functions of microbiomes before and after MC addition on the basis of the levels 2 and 3 pathways in the KEGG database.

### 2.5 Sampling and analysis for various environmental parameters

Various chemical and physical parameters were sampled and measured in the water layers which were consistent with phytoplankton. Temperature was measured via a YSI multiparameter water quality meter (YSI Ltd., USA). The concentrations of Chl *a* were measured via a PHYTO-PAM (Walz, German). After 100 mL of Fuchun River water from each sample was filtered through a GF/F filter membrane (Whatman, Kent, UK), the filtrate was separated into one sterile 15 mL centrifuge tube and one 30 mL glass bottle for inorganic nutrient and dissolved organic carbon (DOC) analyses, respectively. All the samples were frozen and stored at −20°C until further analysis. The concentrations of inorganic nutrients, including nitrate (NO_3_^−^), nitrite (NO_2_^−^), ammonium (NH_4_^+^), silicate (SiO_3_^2−^), and phosphate (PO_4_^3−^), were measured via a SKALAR flow analyzer (Skalar, Breda, Netherlands), and the data quality was monitored via intercalibration (Wang W. T. et al., 2016). DOC concentrations were measured via a total organic carbon analyzer (Analytique Jena, Germany) (Chu et al., 2024).

In all the figures and tables, the samples collected from the surface water and bottom water before MC addition were named Pre. sur and Pre. bot, respectively. The samples collected after MC addition were named After.

## 3 Results and discussion

### 3.1 Effects of MC on phytoplankton community composition

The phytoplankton at the research site can be divided into two categories, namely, eukaryotic and prokaryotic, and prokaryotic phytoplankton refers to cyanobacteria. When cyanobacterial blooms occur, most phytoplankton float on the water surface. Before MC was sprayed, the Chl *a* concentrations in the surface and bottom waters were 180.80 and 43.84 μg L^−1^, respectively. After MC was sprayed, the average Chl *a* concentration in the water was 5.40 μg L^−1^. Based on calculations, the phytoplankton removal efficiency ranged from 87.68–97.01%. The phytoplankton species were identified and analyzed via high-throughput sequencing and bioinformatics analysis. After sequencing, 775,384 raw reads and 545,132 clean reads were produced for prokaryotic phytoplankton analysis (Supplementary Table S1), and 732,416 raw reads and 597,391 clean reads were produced for eukaryotic phytoplankton analysis (Supplementary Table S2).

#### 3.1.1 Cyanobacterial community

**Figure 2:**
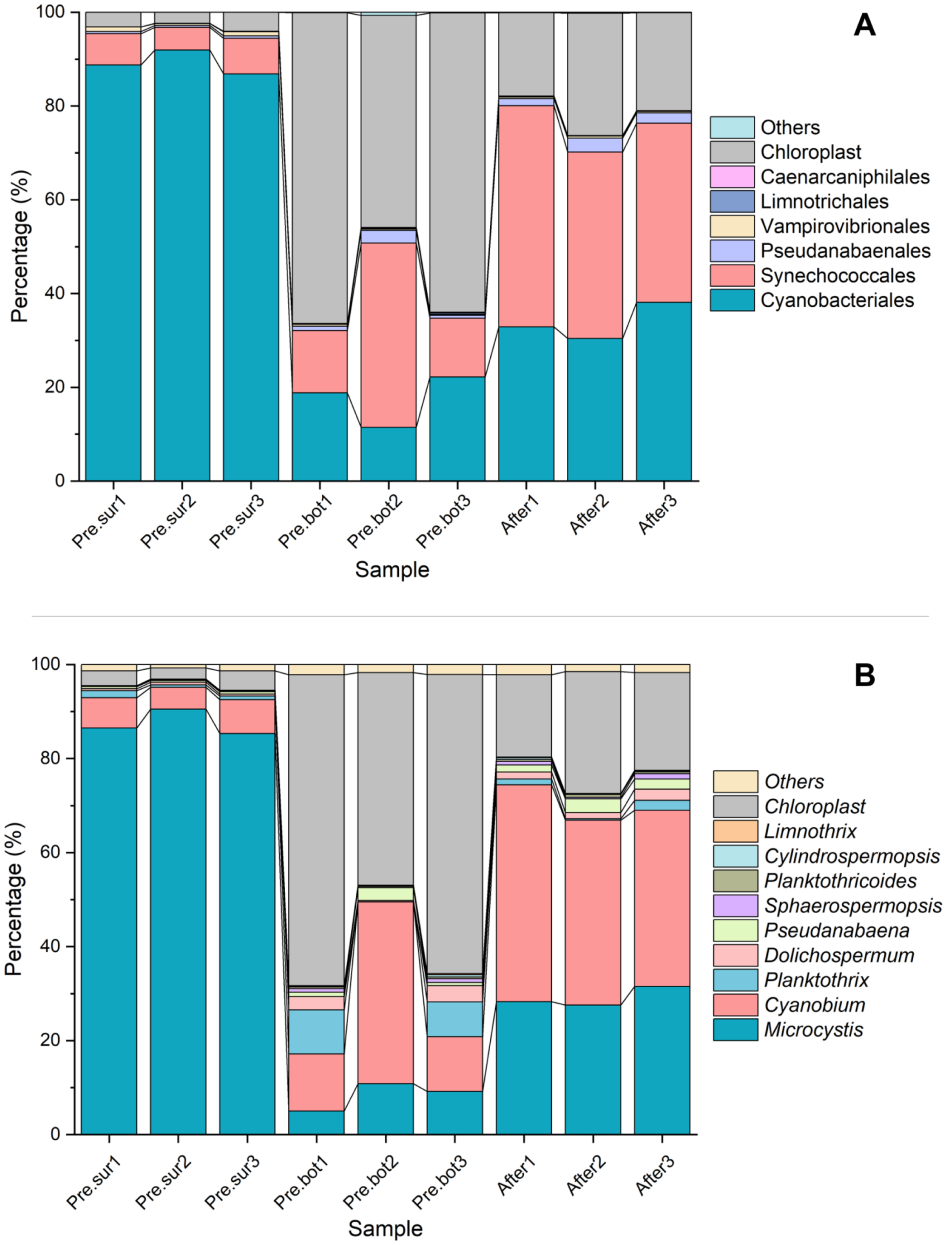
Relative abundance of cyanobacteria in the top 7 orders (A) and the top 10 genera (B) in 9 samples before and after MC treatment.

Cyanophyta was selected from the prokaryotic sequencing data for cyanobacterial community structure analysis. With respect to species composition, at the order level, Cyanobacteriales was dominant in the surface waters, followed by Synechococcales; in the bottom waters, the abundances of both Cyanobacteriales and Synechococcales were relatively high (Figure 2A). Chloroplast DNA has many similarities to the prokaryotic genome, including the ability to encode 16S rRNA. Therefore, the high-throughput sequencing results included chloroplast data that were categorized into the Cyanophyta phylum in the species annotation database. Considering that the number of chloroplasts represents the abundance of eukaryotic phytoplankton to some extent, these chloroplast data were retained in the analysis of cyanobacterial species composition. Figures 2A,B show that the chloroplast abundance in the surface water was less than 5%, which further illustrated that the bloom was caused by cyanobacteria. At the genus level, *Microcystis* sp. was dominant at the surface, followed by *Cyanobium* sp. At the bottom, *Cyanobium* sp. had the highest abundance, followed by *Microcystis* sp. and *Planktothrix* sp. Additionally, *Dolichospermum* sp. and *Pseudanabaena* sp. were present. However, chloroplasts were dominant in the bottom samples, indicating that eukaryotic phytoplankton were abundant. After MC addition, *Cyanobium* sp. became the most abundant taxon in the whole water, with *Microcystis* sp. being the second most abundant taxon, and the abundance of chloroplasts was lower than that at the bottom before MC treatment. MC eliminated the apical dominance of *Microcystis* sp. in the phytoplankton community. These results indicated that MC changed the composition of the cyanobacterial community and that the removal efficiency of different algal species with MC treatment differed.

The dominant species in this study, *Microcystis* sp., is the most common cyanobacterial bloom species and has triggered blooms in many freshwater environments, such as Taihu Lake and Chaohu Lake in China; *Cyanobium* sp., *Planktothrix* sp., and *Dolichospermum* sp. are also common cyanobacterial species (Capelli et al., 2017; Huo et al., 2021). Studies have shown that larger cells with weak electronegativity and strong hydrophilicity on the cell surface more easily aggregate with other particles and flocculate under MC treatment (Vanloosdrecht et al., 1987; Zang et al., 2024). The species belonging to *Microcystis* are unicellular and do not have a sheath covering, and the cells are often aggregated into irregular groups surrounded by extracellular polymeric substances (EPS) that possess gas vacuoles for rapid movement throughout the water column (Hitzfeld et al., 2000; Shen et al., 2011; Yang et al., 2024). In general, EPS provide some protection to algal cells; however, larger colony particles more easily collide with MC particles. In addition, hydrophilic groups such as O-H and P-O-P are contained in EPS, which allow *Microcystis* to more easily flocculate and be removed by MC (Yang et al., 2023). Compared with those of *Synechococcus* sp. (−32.20 mV) and *Anabaena* sp. (−11.00–16.80 mV), the zeta potentials of *Microcystis* species (−11.85–12.07 mV) are weaker (Yang et al., 2023), which also allows *Microcystis* sp. to more easily settle with MC particles. The characteristics mentioned above allow MC to effectively collide with and flocculate *Microcystis* sp. and eliminate the apical dominance of the species. *Cyanobium* sp. are autotrophic picocyanobacteria (Pagliara et al., 2021), which are usually present as solitary or two isomorphic daughter cells after binary fission with little or no evident exterior mucilage (Lee et al., 2020). *Cyanobium* cells tend to escape the effects of MC because their cell size is very small and their surface viscidity is relatively low. These are some of the reasons for the increase in the relative abundance of *Cyanobium* sp. after MC treatment in this study and illustrate that MC has different removal efficiencies for different microalgae. In conclusion, the collision frequency and flocculation efficiency of MC particles for different algal species varied, which altered the relative composition of the phytoplankton.

**Figure 3:**
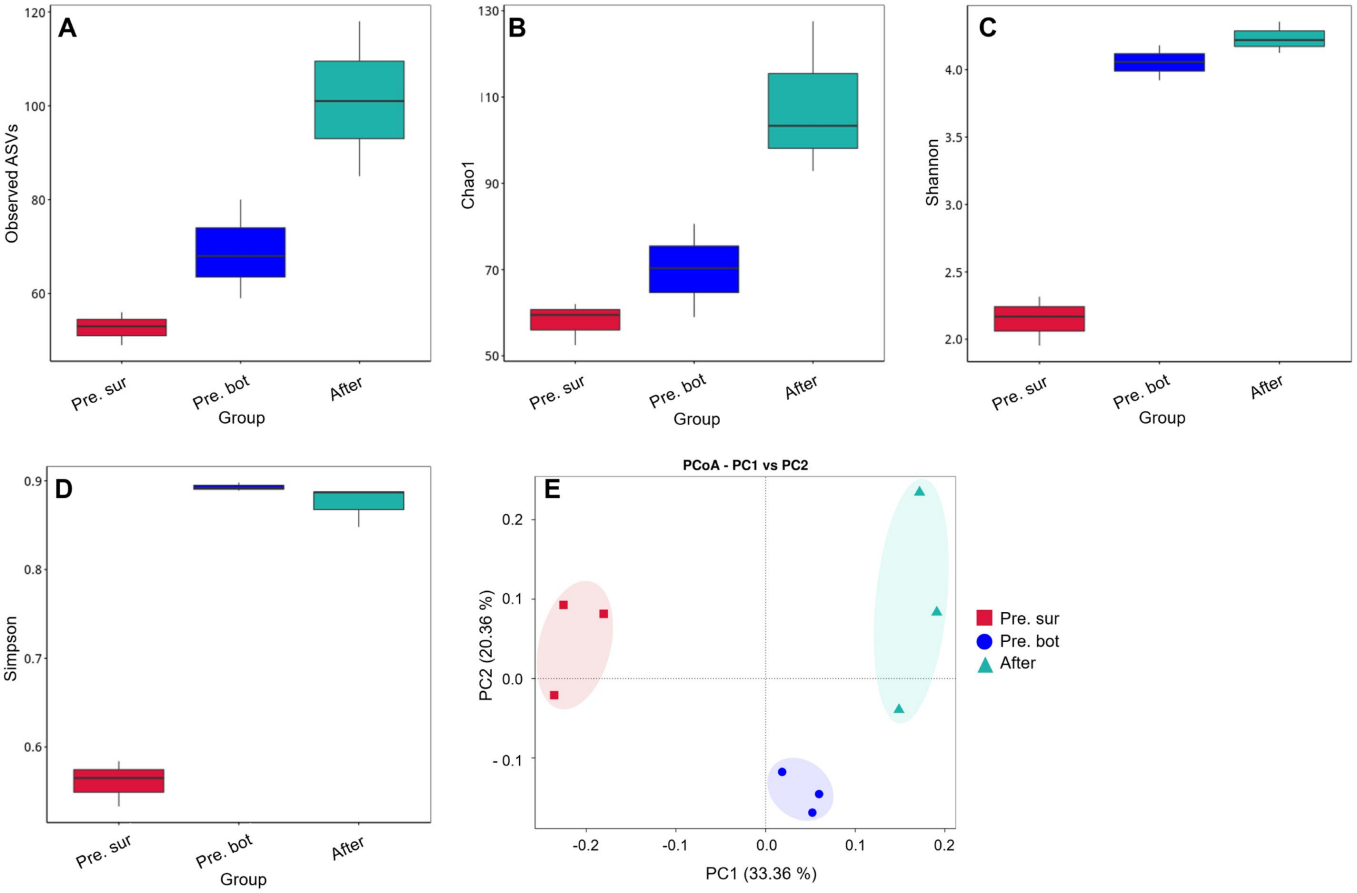
Diversity of the cyanobacterial community. A-D, Alpha diversity indices: Observed ASVs, Chao1, Shannon and Simpson indices, respectively; E, PCoA of cyanobacterial diversity between different groups.

In alpha diversity indices, Observed ASVs refers to the number of directly observed species, and Chao1 refers to the total number of species estimated in a community sample. In addition, the greater the community diversity and the greater the Shannon index are, the greater the species evenness and the greater the Simpson index are. The results of the Observed ASVs and Chao1 index analyses revealed that there were significantly more cyanobacterial species in the bottom water than in the surface water (Figures 3A,B). The values of the Shannon and Simpson indices revealed that the biodiversity of the bottom water was also significantly greater than that of the surface water (Figures 3C,D). Interestingly, the Observed ASVs and Chao1 index increased significantly after MC addition, and the Shannon index increased slightly compared with that in the bottom water before MC addition. These results indicate that MC treatment increased the diversity of the cyanobacterial community. The PCoA results revealed that samples within groups were clustered together (Figure 3E). After MC treatment, the similarity between the samples decreased, indicating that the distribution of residual phytoplankton was nonuniform. These findings are consistent with the results of a previous study carried out in aquaculture seawater (Zhu et al., 2023). In conclusion, MC can mitigate cyanobacterial blooms effectively and eliminate the apical dominance of *Microcystis* sp. Moreover, MC can increase the diversity of cyanobacterial communities, which also helps prevent and control blooms.

#### 3.1.2 Eukaryotic phytoplankton community

**Figure 4:**
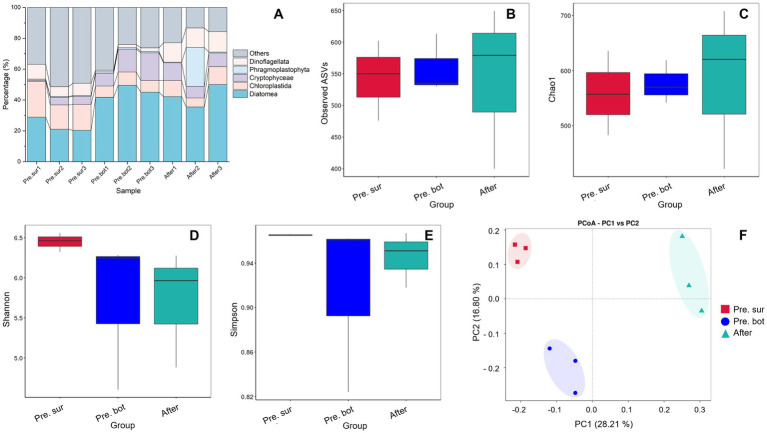
Species relative abundance and diversity of the eukaryote phytoplankton community. A, Relative abundance of eukaryotic phytoplankton in the top 7 phyla; B-E, alpha diversity indices: Observed ASVs, Chao1, Shannon and Simpson indices, respectively; F, PCoA of eukaryote phytoplankton diversity between different groups.

In terms of species composition, the initial community was dominated by diatoms, followed by green algae and cryptophytes (Figure 4A). After MC treatment, diatoms remained the most abundant species, and the relative abundance of dinoflagellates increased (Figure 4A), which may have occurred because dinoflagellates have greater motility and can break free from MC particles. The biodiversity results revealed that the values of the alpha diversity indices Observed ASVs and Chao1 in the surface water were similar to those in the bottom water (Figures 4B,C), indicating that there was no significant difference in the number of eukaryotic phytoplankton species. However, the diversity and evenness of the surface water samples were greater than those of the bottom water samples according to the Shannon and Simpson indices (Figures 4D,E). The PCoA results revealed that samples within groups were clustered together (Figure 4F). After the addition of MC, the Observed ASVs and Chao1 indices increased significantly (Figures 4B,C). MC spraying caused water disturbances, and some algal cells escaped from the MC–algae flocs during the flocculation and settling process (Yu et al., 2017; Zhu et al., 2018), resulting in the mixing of surface and bottom phytoplankton. In addition, MC removed most of the cyanobacterial biomass, which decreased the degree of prokaryotic DNA contamination in the eukaryotic DNA sequencing samples. Both of these factors lead to an increase in the number of detected eukaryotic phytoplankton species. Notably, the Shannon and Simpson indices did not increase significantly after MC treatment. Previous studies in aquacultural waters revealed that the diversity of phytoplankton communities increases under MC treatment (Ding et al., 2021; Zhu et al., 2023). In this study, eukaryotic phytoplankton were not the bloom-forming species. Therefore, MC did not affect eukaryotic communities like elimination of apical dominance. These results suggest that MC has little effect on the community structure of eukaryotic phytoplankton when they do not form blooms. In other words, when the eukaryotic phytoplankton community is healthy, MC treatment is not needed, and MC has no negative effects on the phytoplankton community.

### 3.2 Effects of MC on microbial functions

**Figure 5:**
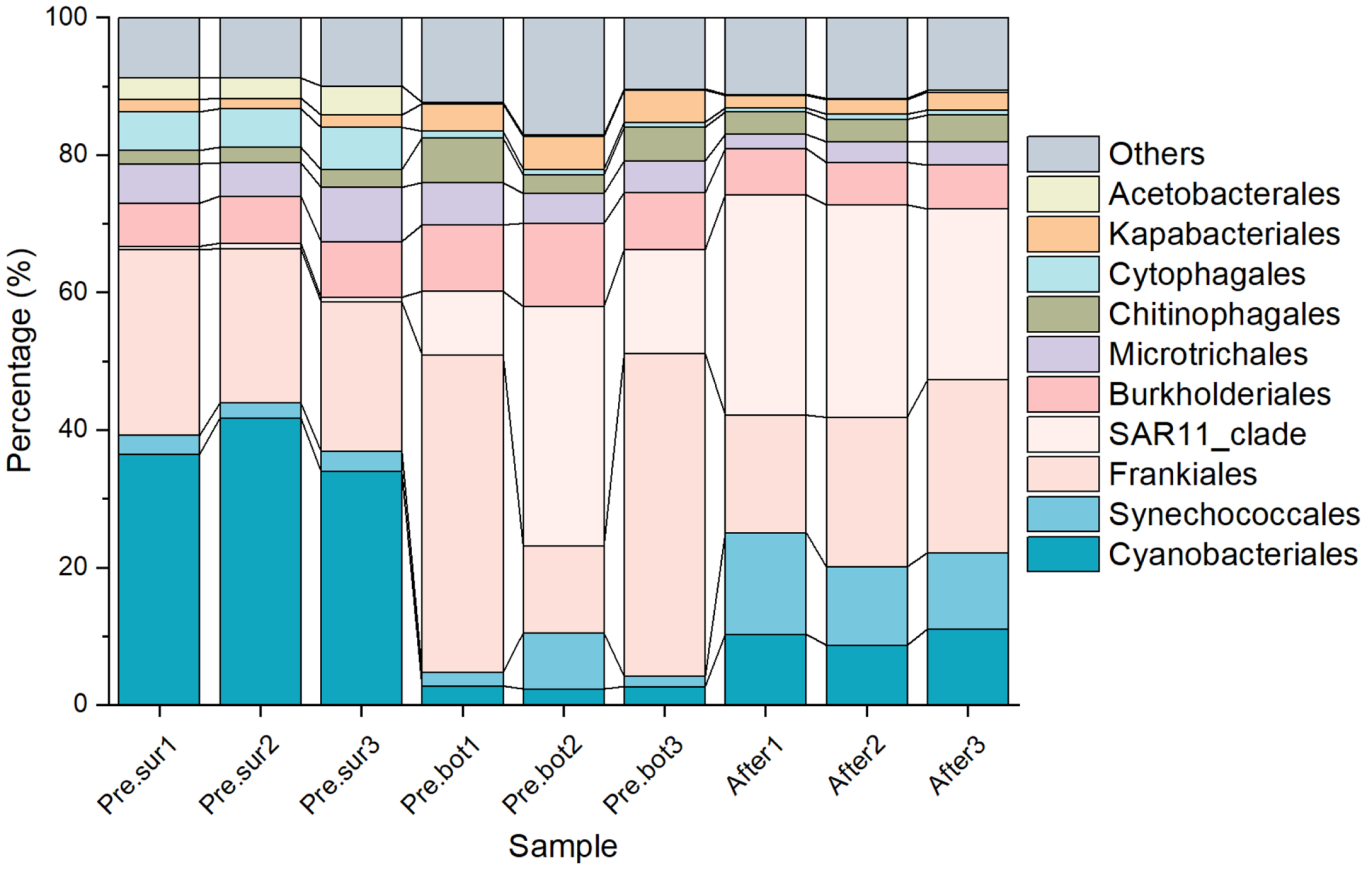
Relative abundance of microbial species in the top 10 orders in 9 samples before and after MC treatment.

**Figure 6:**
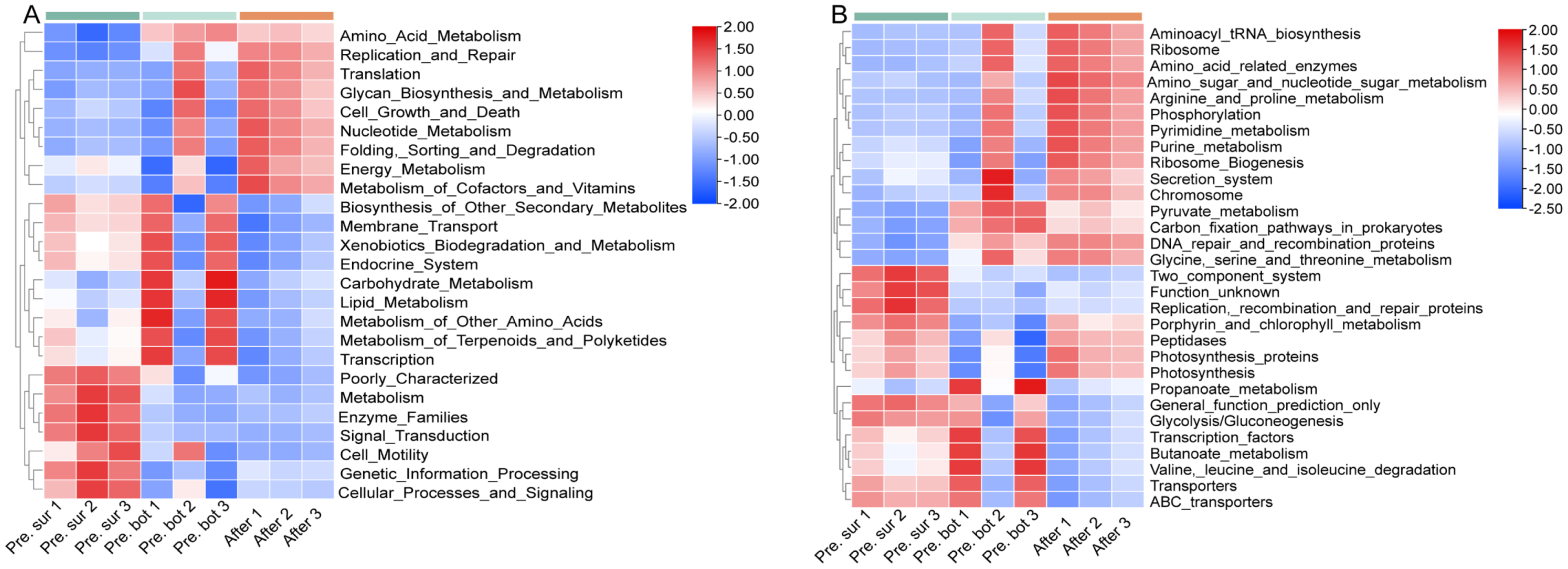
Heatmap of the gene functions of the microbiomes according to KEGG levels 2 (A) and 3 (B).

We studied the effects of MC on the functions of the whole microbiome, which includes cyanobacteria. The species composition results revealed that the abundance of cyanobacteria in surface waters during cyanobacterial blooms accounted for approximately 40% of the microbial community (Figure 5). In addition, the abundance of Frankiales was relatively high in all the samples, which is common in freshwater environments and includes many species with nitrogen-fixing functions (Ruprecht et al., 2021). The relative abundance of the SAR11 clade increased after the addition of MC, and it became the top clade of the prokaryotes. Meanwhile, the relative abundance of cyanobacteria was approximately 20% (Figure 5). The SAR11 clade is excellent in carbon cycling and its dominance may reflect successful adaptation of organisms to resource competition (Salcher et al., 2011). In terms of gene function, the microbial community in the surface water shared some similarities with that in the bottom water before MC treatment, including stronger transport capacity, e.g., membrane transport; stronger synthesis of secondary metabolites, e.g., biosynthesis of other secondary metabolites; and stronger defense and intercellular communication, e.g., metabolism of terpenoids and polyketides. Notably, genes involved in genetic information processing, signal transduction, cellular processes and signaling, cell motility, metabolism, photosynthesis, and enzyme families were abundant in the surface microbial community (Figure 6). This result suggests that the surface microbial community was more capable of cell proliferation, communication, cell migration, metabolism, photosynthesis, and enzyme catalysis, which is consistent with the characteristics of the phytoplankton involved in bloom formation (O'Neil et al., 2012; Yu L. Y. et al., 2023).

After MC treatment, the abundance of cyanobacteria accounted for approximately 25% of the microbial community and genes involved in the level 2 pathways of replication and repair, energy metabolism, glycan biosynthesis and metabolism, cell growth and death, amino acid metabolism, translation, nucleotide metabolism, folding, sorting and degradation, metabolism of cofactors and vitamins were abundant in the residual microbial community (Figure 6A). Among them, the relative abundance of genes encoding DNA repair and recombination proteins involved in replication and repair was significantly greater than that before MC treatment. This result indicates that the residual microbial cells have high potential for DNA damage repair. Previous studies on marine microalgae have shown that MC causes oxidative stress in residual algal cells, leading to DNA damage (Ji et al., 2021; Zhu et al., 2018). Therefore, the residual microbial cells in this study were more likely to survive after being subjected to oxidative stress. Neither *Aureococcus anophagefferens* studies in the laboratory nor phytoplankton community studies in aquaculture water have revealed a significant effect of MC on the glycan biosynthesis and metabolism pathway in the transcriptome (Zhu et al., 2018; Zhu et al., 2023); however, this pathway is enhanced in *Phaeocystis globosa* under low-MC conditions, which favor colony formation (Ren et al., 2022). In this study, the residual microbial community was relatively strong in terms of glycan biosynthesis and metabolism, and secretion system. This result suggests that the residual microbiome has the potential to aggregate and form cell clusters in the residual microbial community. In addition, the residual microbiome had a relatively high abundance of genes involved in energy metabolism, including carbon fixation pathways in prokaryotes and photosynthesis, as well as pathways related to amino acid, nucleotide, and vitamin metabolism. Therefore, the residual microbiome is excellent in metabolism and can provide nutrients to the phytoplankton promoting growth. The results of physiological and transcriptomic studies of marine microalgae also revealed that the expression of genes involved in metabolism was generally increased, the expression of photosynthesis genes was upregulated, and photosynthesis system II was activated in residual cells in response to the effects of MC (Liu et al., 2018; Zhu et al., 2018). These reports are consistent with the functional changes in the microbial communities observed in this study. Oppositely, the abundance of genes involved in genetic information processing, signal transduction, and photosynthesis was decreased indicating that the residual microbiome was week in proliferation and light energy harvesting. In summary, the results of the PICRUSt analysis revealed that the residual microbial community had greater abilities to repair and metabolize carbon, greater carbon fixation and cycling advantages, and the potential to aggregate and form colonies after the addition of MC, which was favorable for survival and competitive growth in a stressful environment.

### 3.3 Mechanisms by which MC controls *Microcystis* sp. blooms

**Figure 7:**
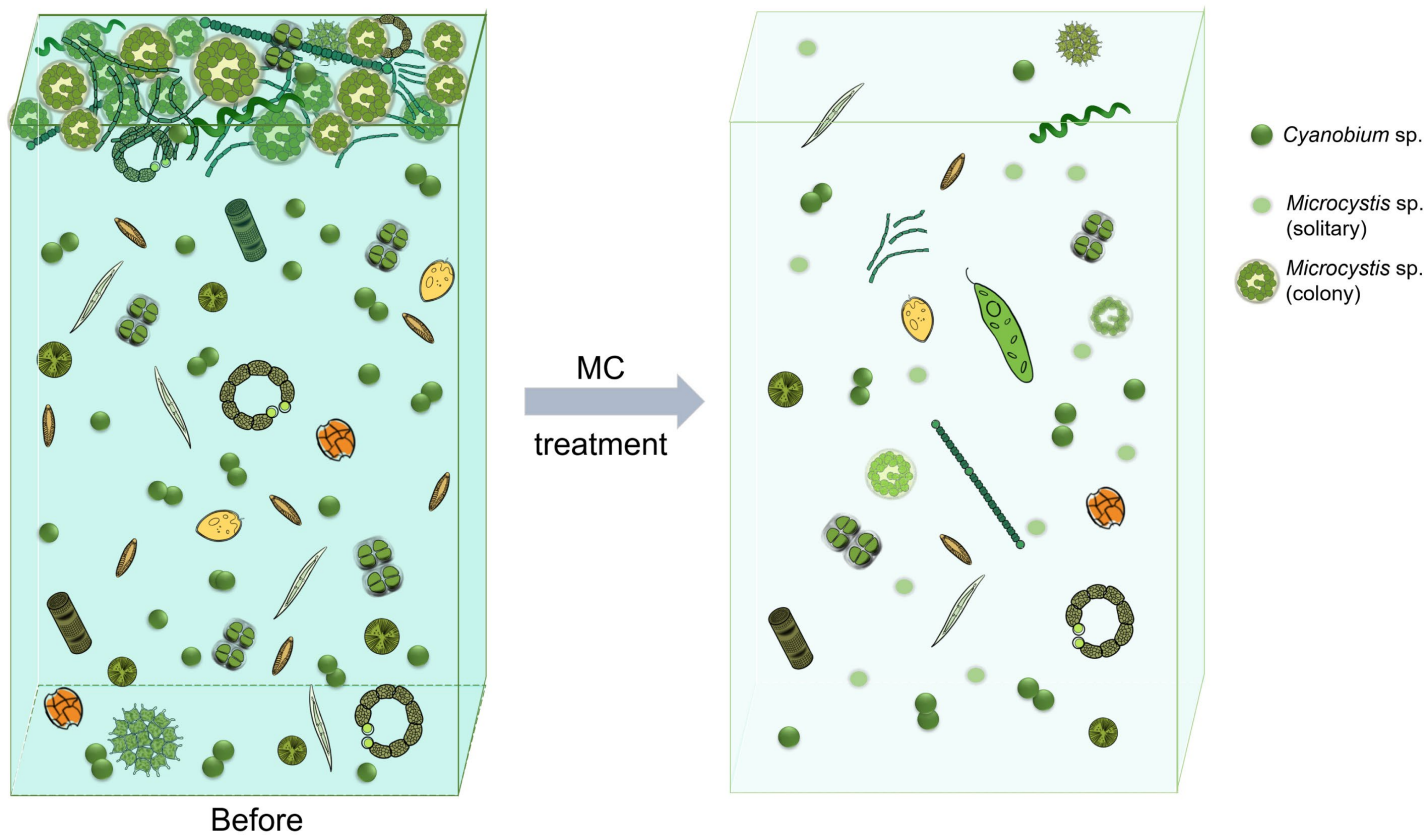
Schematic showing the changes of the phytoplankton community composition under modified clay treatment.

An abnormal increase in phytoplankton biomass provides a visual indicator of bloom formation. The direct effect of MC in controlling HABs involves removing bloom biomass from water through flocculation (Jiang et al., 2021; Liu et al., 2016). In this study, the phytoplankton biomass in the water decreased by approximately 90% under MC treatment (Figure 7). These phytoplankton combine with MC particles to form flocs and settle from surface waters to the bottom where cells cease to grow or die (Guenther and Bozelli, 2004). The results revealed that the relative abundance of the dominant bloom-formation species *Microcystis* sp. also decreased significantly after the addition of MC (Figure 3B), further indicating that MC removed most of the *Microcystis* sp. from the water and eliminated the “apical dominance” of the species (Figure 7). Previous studies have shown that a 70–80% removal efficiency of algae biomass is sufficient to control HABs. Notably, after MC treatment, residual cells (20–30% of the initial cell density) rarely continue to grow or form blooms (Yu et al., 2017). What are the mechanisms by which MC controls blooms caused by *Microcystis* sp. colonies?

*Microcystis* sp. can aggregate and form EPS that surrounds cell groups called colonies, which are the most common form during blooms (Wu et al., 2020; Yang et al., 2024). Therefore, the formation of colonies is crucial to bloom formation. The ability of *Microcystis* sp. to form colonies is similar to that of some marine microalgae in the genus *Phaeocystis*, such as *P. globosa*, which frequently blooms in the South China Sea (Wang et al., 2021). MC has been widely used in the mitigation of marine HABs, including *P. globosa* blooms (Yu Z. M. et al., 2023). Qiu et al. (2020) developed an oxidized composite MC (OXI-MC), the important component of which was the PMS used in this study, to increase the removal efficiency of *P. globosa* blooms and destroy the mucilaginous envelope of the colonies. The OXI-MC can reduce the content of extracellular polysaccharides in *P. globosa* cultures, destroy the mucilaginous envelope around colonies, and inhibit colony formation (Qiu et al., 2020). Another study revealed that MC caused oxidative stress in residual cells, preventing these cells from reaching a high biomass (Ren et al., 2022). Owing to the limitations of the field study conditions, we could not obtain microscopy images but observed that the diameter of the *Microcystis* colonies decreased and that the number of solitary cells increased significantly under MC treatment. This phenomenon indicates that MC can destroy the EPS envelope of *Microcystis* colonies while reducing their biomass. The observed effects of MC on *P. globosa* suggest that MC also causes oxidative stress and inhibits the formation and growth of *Microcystis* colonies. In addition, it has been reported that the morphological changes that occur between colonies and solitary cells in *Microcystis* sp. are the result of interactions with heterotrophic bacteria (Shen et al., 2011; Van Le et al., 2022). There are also significant differences in phycosphere bacterial composition and function between colonies and solitary cells of *P. globosa* strains (Zhu et al., 2024). Both the results of this study (Figure 4) and those of a previous report (Song et al., 2023) revealed that MC changed the structure of phycosphere bacterial communities, which affected the physiological activities of microalgae. Overall, the removal of bloom biomass, direct destruction of *Microcystis* colonies, changes in the structure of bacterial communities, and potential oxidative stress to residual cells inhibited growth and colony formation by *Microcystis* sp., revealing the mechanism by which MC controls these blooms.

**Figure 8:**
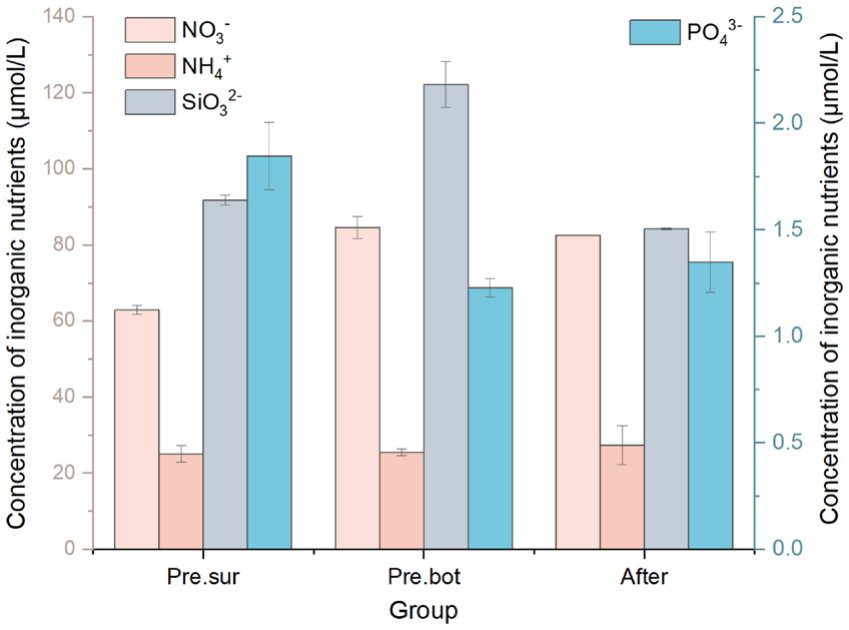
Effects of modified clay on different nutrients.

The effects of nutrients on the phytoplankton community were also considered. We monitored the concentrations of nutrients in the water before and after MC addition. Nitrogen, phosphorus, and silica are important biogenic elements for phytoplankton growth. The results revealed that before MC addition, the nitrate concentration in the surface water was 62.87 μmol/L, which was significantly lower than the 84.58 μmol/L observed in the bottom water. The concentrations of ammonium were 25.01 μmol/L and 25.42 μmol/L in the surface and bottom water, respectively. Like nitrate, the silicate concentration in the surface water was 91.76 μmol/L, which was significantly lower than the 122.20 μmol/L observed in the bottom water (Figure 8). However, the concentrations of phosphate in the water were lower, at 1.85 μmol/L and 1.22 μmol/L in the surface and bottom water, respectively. In addition, the concentration of dissolved organic nitrogen (DON) in the bottom water was 4.84 μmol/L, and the concentration of dissolved organic phosphorus (DOP) in the bottom layer was 3.94 μmol/L. Therefore, nitrogen in the water body was dominated by inorganic forms, whereas phosphorus was dominated by organic forms. Under these nutrient conditions, diatoms have no competitive advantage over cyanobacteria even when silicate is abundant (O'Neil et al., 2012). After MC addition, the concentration of nitrate was 82.57 μmol/L, and the ammonium concentration was 27.39 μmol/L, which did not significantly change from the values before MC addition; however, the concentration of silicate was reduced to 84.22 μmol/L. These results are consistent with the results of previous reports on the effects of MC on water quality (Chi et al., 2022; Zhu et al., 2023). The concentration of DON increased by approximately 20% after MC addition. Additional DON may have been released from the lysed algal cells under MC treatment. However, there were no significant changes in the concentrations of phosphate (1.34 μmol/L) or DOP (3.71 μmol/L) (Figure 8). Therefore, there was no significant change in the N/P ratio under MC treatment in this study, and the Si/N and Si/P ratios were significantly reduced. However, laboratory studies have shown that MC can remove phosphorus, leading to an increase in the N/P ratio (Lu et al., 2017; Zhu et al., 2019). In the field, increasing N/P ratios and decreasing Si/N and Si/P ratios also occur under MC treatment (Chi et al., 2022, Zhu et al., 2023). In this study, the sum of the phosphate and DOP concentrations was <6 μmol/L, which was lower than that reported in previous studies. The results of Chi et al. (2022) also revealed that the removal of phosphorus by MC was not significant during the prefarming period when the TOP concentration was <10 μmol/L. In conclusion, the changes in nutrient levels caused by MC treatment were not sufficient to inhibit phytoplankton growth, and there was still a risk of bloom formation.

Maintaining the stability of the phytoplankton community is a precondition for avoiding blooms. In this study, diatoms dominated the eukaryotic phytoplankton community; however, their growth rates were lower than those of cyanobacteria at higher temperatures (> 25°C) (O'Neil et al., 2012). After MC addition, the relative abundance of diatoms remained highest in the eukaryotic phytoplankton community (Figure 4A), but the potential for diatom bloom formation decreased with decreasing Si/N and Si/P ratios. The composition of the cyanobacterial community also changed significantly under MC treatment, with *Cyanobium* sp. replacing *Microcystis* sp. as the most abundant species (Figure 2). This process is similar to the elimination of apical dominance in plants, which promotes lateral bud growth. Therefore, *Microcystis* lost its dominance in the phytoplankton community and *Cyanobium* had the opportunity to occupy more ecological niches to compete with it after the addition of MC. In addition, species in *Cyanobium* can live symbiotically or epiphytically with other algae, such as *Ochromonas,* or with organic particles, and have a strong capacity for organic matter uptake and utilization (Jezberová and Komárková, 2007). In addition, *Cyanobium* sp. respond hormetically to increased ammonia levels with no growth inhibition at relatively high concentrations (Alquezar and Anastasi, 2013). In the water at the research site, phosphorus was dominated by organic forms, and the concentration of DON increased after MC addition, which favored *Cyanobium* sp. growth. Moreover, microcystins produced by *Microcystis* can inhibit the growth of other algal species, such as *Cyanobium*. In contrast, the destruction of *Microcystis* colonies increases the vulnerability of the cells to predation by *Ochromonas* (Wei et al., 2022). Temperature is a crucial factor in regulating the phytoplankton community, among which *Cyanobium* sp. prefer relatively low temperatures, and *Microcystis* sp. blooms are prone to form in waters with relatively high temperatures. The water temperature was 28.4°C during the experiment, which is favorable for *Microcystis* growth. In addition, the biodiversity of the cyanobacterial community increased under MC treatment (Figures 2A–D), which is beneficial for maintaining the stability of the phytoplankton community. In conclusion, increased phytoplankton diversity, elimination of the apical dominance of *Microcystis* by MC, predation by other phytoplankton, and competition between *Cyanobium* and *Microcystis* prevent the reoccurrence of blooms with MC treatment.

## 4 Conclusion

On the basis of the experiments in which MC controls cyanobacterial blooms in the Fuchun River, we draw the following conclusions: (1) MC can rapidly remove most of the cyanobacterial bloom-forming biomass from freshwater; (2) MC eliminates the apical dominance of *Microcystis* sp. in the phytoplankton community and changes the cyanobacterial community structure; it also enhances competition between *Cyanobium* and *Microcystis* and increases cyanobacterial community diversity; however, the change in the eukaryotic phytoplankton community structure was not significant under MC treatment, and had no negative effects on the healthy eukaryotic phytoplankton community; (3) MC can destroy *Microcystis* colonies and change the phycosphere microbiome, hindering bloom development. These findings illustrate the mechanisms by which MC controls cyanobacterial blooms and indicate that MC constitutes an environmental friendly technology to mitigate blooms in freshwater. It was also concluded that under MC treatment, residual microorganisms, including cyanobacteria, have stronger antioxidant and metabolic capabilities and have the potential to aggregate and form colonies. In addition, in the high nitrogen and low phosphorus water, the nutrient level changes caused by MC are not sufficient to reduce the risk of blooms. The experimental area is a shipping channel that needs to be opened periodically, making it impossible to close the water for long periods. The lack of prolonged monitoring is a limitation of this study. We plan to conduct more in-depth and comprehensive studies in the future.

## Data Availability

The datasets presented in this study can be found in online repositories. The names of the repository/repositories and accession number(s) can be found at: https://www.ncbi.nlm.nih.gov/genbank/, SUB14762309 and https://www.ncbi.nlm.nih.gov/genbank/, SUB14769304.

## Footnotes

^1^http://ccb.jhu.edu/software/FLASH
