# Supplementary material for: Double effects of mitigating cyanobacterial blooms using modified clay technology: regulation and optimization of the microbial community structure
Source: Front Microbiol. 2024 Nov 5;15:1480069. doi: 10.3389/fmicb.2024.1480069 (PMC11573764; doi:10.3389/fmicb.2024.1480069)
Supplement: Supplementary file 1 [file Table_1.docx]

**Table S1. The data production of 16S rDNA sequencing of bacteria including cyanobacteria**

| **Sample** | **Raw reads** | **Effective Tags** | **Effective**  **bases (nt)** | **Average**  **length (nt)** | **GC (%)** | **Q20 (%)** | **Q30 (%)** | **Effective percentage (%)** |
| --- | --- | --- | --- | --- | --- | --- | --- | --- |
| **Pre.sur1** | 85,701 | 61,354 | 25,170,915 | 410.26 | 54.37% | 98.69% | 95.59% | 71.59% |
| **Pre.sur2** | 86,128 | 61,223 | 25,132,252 | 410.50 | 54.29% | 98.65% | 95.47% | 71.08% |
| **Pre.sur3** | 87,335 | 61,914 | 25,433,890 | 410.79 | 54.39% | 98.61% | 95.39% | 70.89% |
| **Pre.bot1** | 92,650 | 67,754 | 27,917,135 | 412.04 | 52.90% | 98.67% | 95.48% | 73.13% |
| **Pre.bot2** | 92,940 | 65,288 | 26,869,314 | 411.55 | 51.44% | 98.17% | 94.30% | 70.25% |
| **Pre.bot3** | 91,433 | 62,971 | 25,909,218 | 411.45 | 52.78% | 98.10% | 94.15% | 68.87% |
| **After1** | 88,573 | 61,768 | 25,263,151 | 409.00 | 51.79% | 98.61% | 95.30% | 69.74% |
| **After2** | 63,911 | 40,534 | 166,028,73 | 409.60 | 51.71% | 98.21% | 94.35% | 63.42% |
| **After3** | 86,713 | 62,326 | 25,551,115 | 409.96 | 52.17% | 98.57% | 95.18% | 71.88% |

**Table S2. The data production of 18S rDNA sequencing of phytoplankton**

| **Sample** | **Raw reads** | **Effective Tags** | **Effective**  **bases (nt)** | **Average**  **length (nt)** | **GC (%)** | **Q20 (%)** | **Q30 (%)** | **Effective percentage (%)** |
| --- | --- | --- | --- | --- | --- | --- | --- | --- |
| **Pre.sur1** | 64,519 | 48,436 | 14,730,407 | 304.12 | 45.58 | 98.84 | 95.83 | 75.07% |
| **Pre.sur2** | 69,227 | 56,076 | 17,117,486 | 305.26 | 45.34 | 98.90 | 95.98 | 81.00% |
| **Pre.sur3** | 94,680 | 73,517 | 22,331,919 | 303.77 | 45.01 | 98.82 | 95.71 | 77.65% |
| **Pre.bot1** | 87,374 | 66,622 | 20,392,517 | 306.09 | 45.70 | 98.64 | 95.13 | 76.25% |
| **Pre.bot2** | 58,550 | 46,051 | 14,123,867 | 306.70 | 45.38 | 98.82 | 95.73 | 78.65% |
| **Pre.bot3** | 92,755 | 78,682 | 24,085,445 | 306.11 | 47.01 | 98.74 | 95.41 | 84.83% |
| **After1** | 88,252 | 75,776 | 23,321,289 | 307.77 | 44.46 | 98.71 | 95.32 | 85.86% |
| **After2** | 88,402 | 77,785 | 23,985,529 | 308.36 | 46.23 | 98.38 | 94.28 | 87.99% |
| **After3** | 88,657 | 74,446 | 22,837,625 | 306.77 | 44.17 | 98.90 | 95.95 | 83.97% |
